# Financial concerns and psychological distress among Asian Americans during the COVID-19 pandemic: the moderating role of benefit finding and received pay

**DOI:** 10.3389/fpubh.2025.1706398

**Published:** 2026-02-02

**Authors:** Sabreet Kaur Dhatt, Michael P. Huynh, Erika Mey, Lan N. Ðoàn, Kris Pui Kwan Ma, Anne Saw

**Affiliations:** 1 Department of Psychological and Brain Sciences, University of Wisconsin-Milwaukee, Milwaukee, WI, United States; 2 Department of Public Health, California State University San Marcos, San Marcos, CA, United States; 3 Department of Health, Society, and Behavior, Wen School of Population and Public Health, University of California, Irvine, Irvine, CA, United States; 4 Department of Population Health, New York University Langone Health, New York, NY, United States; 5 Department of Family Medicine, University of Washington School of Medicine, Seattle, WA, United States; 6 Department of Psychology, DePaul University, Chicago, IL, United States

**Keywords:** Asian Americans, benefit finding, COVID-19, financial concerns, psychological distress

## Abstract

**Introduction:**

The potential buffering role of benefit finding, a cognitive and behavior adaptation process, in the relationship between financial concerns and psychological distress is not well understood among Asian American populations. Our study examined how financial concerns impacted Asian Americans’ mental health, specifically in the presence of benefit finding and received pay during the COVID-19 pandemic.

**Methods:**

Survey-weighted data from the 2021 Asian American and Native Hawaiian/Pacific Islander COVID-19 Needs Assessment Project was analyzed to test the associations between financial concerns and psychological distress among Asian American adults 18 years or older (unweighted N = 3,152). Multivariable linear regression models included an interaction term for benefit finding, financial concerns, and received pay to test the moderation effect of benefit finding on psychological distress.

**Results:**

Reporting financial concerns increased the likelihood of psychological distress (*β*: 1.24, 95% CI: 0.91, 1.56). Asian Americans who reported greater levels of benefit finding had lower psychological distress, but benefit finding alone did not moderate the relationship between financial concerns and psychological distress. However, having both greater levels of benefit finding and received pay protected Asian American participants the most from psychological distress when experiencing financial concerns.

**Discussion:**

Our findings suggest that benefit finding and received pay combined may have important implications for interventions and policy-level changes targeting financial concerns among Asian Americans. Future research should explore the relationships between benefit finding and health outcomes over the life course, other protective coping responses for Asian Americans, and potential differences by Asian ethnicity or specific subgroups.

## Introduction

The emergence of the COVID-19 pandemic marked a profound global health crisis, disrupting daily life (e.g., quarantine and social distancing protocols) and imposing significant stressors on individuals and communities. However, the burden of the pandemic was not borne equally across communities. Minoritized racial and ethnic groups, particularly Asian Americans, experienced a disproportionate share of the pandemic’s adverse effects. COVID-19 exacerbated existing stressors and social determinants of health for Asian Americans, including access to health and mental health care, which increased the vulnerability to the disease, hospitalization, and death (1–4). Occupational and community-level factors further compounded these risks. Asian Americans’ disproportionate representation in healthcare, transportation, and service industries, as well as proximity to community members in these occupations, increased Asian Americans’ exposure to infection (5, 6). Asian Americans were also vulnerable to COVID-19 exposure and infection outside of work. Cultural factors, such as living in three-generation households, made it challenging for Asian Americans to practice social distancing (6, 7). Moreover, inadequate access to linguistically and culturally responsive care marginalized specific Asian American subpopulations, including adults who spoke a primary language other than English and older adults. Notably, over one-third of Asian Americans have limited English proficiency (LEP), and one in five Asian American households is linguistically isolated (8). Older Asian Americans with LEP were more likely to encounter social isolation and abandonment by the healthcare system due to comorbidities and significant language barriers (9). Additionally, Asian immigrant populations were at increased risk for poorer COVID-19 outcomes due to transportation barriers to accessing care, limited access to COVID-19 testing, risk-avoidant behaviors (e.g., staying home), and fear of being deemed a public charge (9–12). Together, the intersection of health-related disparities and structural barriers compounded the vulnerability of Asian American communities during the pandemic.

### Financial concerns

Asian Americans also experienced economic stressors during the COVID-19 pandemic that included unemployment, discrimination against Asian-owned businesses, and the loss of food businesses (13). The unemployment rate for Asian Americans increased from 2.8% in 2019 to 15% in May 2020; and, as of April 2020, Asian American adults were 5.4% more likely to lose employment than White individuals in April 2020 (14). While unemployment rates increased across all racial groups, Asian Americans were more impacted by the COVID-19 lockdown period than other racial groups; Asian American individuals with low education levels were most impacted and were more likely to lose employment and were less likely to regain employment than their White counterparts (15). Additionally, the sharp rise in xenophobia and anti-Asian hate resulted in decreased consumer traffic in Asian restaurants and the closures of Asian-owned businesses, including produce vendors and restaurants in dense Asian ethnic neighborhoods (13, 16, 17). These economic challenges experienced by Asian American communities were also aggravated by increases in unprovoked violence, vandalism, and discrimination against people racialized as Asian Americans (18–20). These experiences have shown to be associated with significantly higher odds of depression, anxiety, non-suicidal self-injury, suicidal ideations, and binge drinking (21, 22).

### Psychological distress

Asian people had significantly higher psychological distress associated with permanent job loss, temporary unemployment, and pay cuts during the pandemic compared to other racial groups (23). Among Asian Americans, Filipino and Vietnamese adults experienced the highest prevalence of financial hardship during COVID-19; these financial hardships have been associated with anxiety and hopelessness (24). Asian Americans experienced a greater mental health impact during the pandemic, with a 2.04 times increase in depression between 2019 and 2020, whereas White, Black, and Hispanic individuals did not show this difference (25). Among Asian subgroups, South Asian Americans were shown to have significantly higher odds of psychological distress than non-Hispanic White Americans (26).

Our study is guided by the transactional model of stress and coping, proposed by Lazarus and Folkman, which explains the relationship between the individual, stressful environment, and individual’s appraisal of the environment (27, 28). This model posits that individuals first assess the threat level of their environment and then formulate a coping response (27–29). The coping tasks of regulating emotions and directly managing the stressful situation both rely on an individual’s appraisal of available internal and external resources to aid them (27–29). Research has demonstrated that culturally-influenced coping among Asian Americans, such as having social support, moderated the relationships between COVID-19-related discrimination and psychological well-being among Asian American communities (30). Moreover, such culturally relevant coping strategies allowed Asians to experience positive effects, such as providing a sense of belonging to one’s community (78).

### Benefit finding

Current research on the COVID-19 pandemic’s impacts on Asian American mental health primarily focuses on negative effects; however, these findings may not reflect the full range of responses to stressful events. In addition to survival response and recovery, thriving is another possible outcome following stressful situations and is associated with posttraumatic growth and positive change (31). Positive changes may include resonating with others, personal strength, and a greater appreciation of life (32). These positive effects following a traumatic life event are understood as benefit finding (33). Benefit finding is a cognitive and behavioral adaptation process in which individuals perceive personal, social, psychological, and spiritual benefits from adverse life experiences, and it is related to resilience, posttraumatic growth, and positive mental health outcomes (33–36). This adaptive pathway is a meaning-making process that allows individuals to identify benefits from adversity (37, 38).

Benefit finding is related to, but distinct from, constructs such as positive reappraisal coping and posttraumatic growth. Positive reappraisal coping is an effortful strategy that involves deliberately and repeatedly interpreting stressful experiences in a favorable light (38). In contrast, benefit finding is a process in which individuals naturally identify benefits from adversity without sustained cognitive reappraisal (38). Posttraumatic growth, on the other hand, refers to a lasting positive transformation that arises from a life crisis (38). Unlike posttraumatic growth, benefit finding can occur in response to a broad range of stressors and be experienced soon after an adverse life event (35). As such, benefit finding represents a unique mechanism through which individuals can adapt to adversity and experience resilience and positive mental health outcomes (33–36).

Benefit finding functions as a form of meaning-focused coping, where individuals draw upon their beliefs, values, and goals to cope during a stressful period (39). In the context of the COVID-19 pandemic, we frame benefit finding as a coping mechanism that can influence the appraisal process between financial concerns and mental health consequences. The pressures to succeed and strive for career paths have been shown as a salient theme affecting the mental health of Asian Americans (40), suggesting that risks to income and financial stability may be a key source of stress in this population. Stressors and coping mechanisms that are particularly salient for Asian Americans may come from interdependent networks. While factors such as filial piety are not unique to Asian cultures (41), Asian Americans are still largely shaped in their development by values of collectivism and interdependence (41). This means that a grounding in finding appreciation in life and gratitude for others through benefit finding may be beneficial for Asian Americans when coping with stressors. At the same time, because of the limited research that directly assesses benefit finding in the stressor-health pathway among Asian Americans, this brings questions as to what extent benefit finding influences the appraisal process between financial concerns and mental health outcomes.

Several studies have examined positive responses, such as benefit finding, to the COVID-19 pandemic. For example, a study on undergraduate students and employees revealed that despite experiencing negative impacts of the pandemic, such as increased fear and stress, participants demonstrated resilience, increased appreciation for life, improved relationships, and better well-being during the pandemic (42). A study in China examined students’ perspectives on home quarantining during the pandemic and identified benefit finding as a critical protective factor against adverse mental health outcomes, including depression and stress, during the pandemic (43). Similarly, a study found that benefit finding was an adaptive coping strategy among college students in the United States during COVID-19; specifically, students who engaged in benefit finding, such as expressing gratitude, were less likely to exhibit feelings of stress, apprehension, anxiety, and psychological distress (44). Furthermore, interventions that engage in benefit finding, such as writing about positive thoughts and emotions experienced during the COVID-19 pandemic, have decreased anxiety and depression in adults between the ages of 20 and 65 years (45). Emerging longitudinal research found that Asian Americans were more likely to report benefit finding during the COVID-19 pandemic than White counterparts, particularly among individuals experiencing greater distress and stronger social support, with modest but significant associations with well-being (46). Although one study of Asian and Mexican American breast cancer survivors, conducted pre-pandemic, suggests that benefit finding is a culturally salient coping strategy (47), there has been a dearth of studies examining benefit finding among Asian Americans during the COVID-19 pandemic.

### Current study

Early in the COVID-19 pandemic, unemployment rates in the United States reached unprecedented levels. The unemployment rate surged from 4.4 to 14.7% by April 2020, more than tripling during that period (48). Given that deaths pertaining to COVID-19 became an increasing concern during this time, it was difficult for people to experience benefit finding, even more so for individuals who were unemployed (42). Individuals who were unemployed were more likely to experience adverse mental health conditions, including stress and depression (48). Therefore, it is probable that individuals with a stable source of income were not as stressed or anxious during this time. Although benefit finding may be associated with improved mental well-being and greater appreciation for life, this was not consistent with the involvement of financial concerns, physical health problems, and mental health conditions (49). Therefore, benefit finding was not as evident or strong among individuals who did not receive pay during the pandemic.

The COVID-19 pandemic negatively impacted many Asian Americans due to a steep decrease in employment rates (15, 50). Compared to other racial and ethnic groups, Asian Americans were more likely to lose their jobs (15). However, Asian Americans who experienced the highest rates of unemployment were those who did not have a high educational attainment (15). Consequently, it is important to explore the relationship between benefit finding and received pay in relation to the pandemic among Asian Americans. We frame received pay as an example of instrumental or tangible support, which is a form of practical assistance to help others (51). The transactional model of stress and coping proposes how tangible support may act as a buffer between stressors and health outcomes (28), which suggests that received pay may be one mechanism to reduce the harmful effects of financial concerns on psychological distress.

#### Research gaps and questions

Despite extensive research on the negative mental health impact of the COVID-19 pandemic on Asian Americans, few studies have examined the potential protective role of benefit finding in this population. While benefit finding has been identified as a culturally salient coping strategy in pre-pandemic studies and among other populations during COVID-19, there is a lack of research investigating how Asian Americans engaged in benefit finding in response to financial stressors during the pandemic. Furthermore, to our knowledge, prior studies have not examined how tangible means of support, such as receiving pay, may interact with benefit finding to buffer psychological distress. This gap limits our understanding of culturally relevant coping strategies that may inform interventions aimed at mitigating mental health disparities in Asian American communities.

To this end, we examined financial concerns during the pandemic to understand how this stressor impacted psychological distress among Asian American adults in the presence of benefit finding, a cognitive and behavioral adaptation process in response to stressful events. The present study focused on answering the following research questions: (1) What is the relationship between financial concerns and psychological distress? (2) Does benefit finding moderate the association between financial concerns and psychological distress? (3) Is there evidence for both benefit finding and received pay to moderate the relationship between financial concerns and psychological distress? We hypothesized that, as a meaning-focused coping mechanism, benefit finding will moderate the association between financial concerns and psychological distress in Asian Americans during the COVID-19 pandemic. We examine receiving pay as a main analytic variable to determine whether the combined effects of benefit finding and received pay would be more beneficial to mental health than benefit finding alone. We hypothesized that individuals with both greater levels of benefit finding and received pay, in the presence of financial concerns during COVID-19, will have lower levels of psychological distress compared to people with lower levels of benefit finding and no received pay. Understanding the range of responses to adverse life events may inform culturally relevant protective factors and coping strategies that may alleviate the ongoing effects and widespread financial concerns of the COVID-19 pandemic in Asian American communities.

## Materials and methods

### Dataset and sample

We analyzed data from the Asian American and Native Hawaiian/Pacific Islander (AA & NH/PI) COVID-19 Needs Assessment Study, a survey administered by the Asian American Psychological Association between January 18 and April 9, 2021. Informed consent was obtained from participants at the beginning of the survey, and participants were paid through a $10 gift card or equivalent compensation for panel participants. Further information about the study design and sample characteristics are detailed elsewhere (52, 53). Our sample included participants who self-identified as Asian American. Briefly, the AA & NH/PI COVID-19 Needs Assessment (N = 5,130) included 3,975 Asian American and 1,267 NH/PI adults 18 years and older. The survey was available in nine Asian languages (Bangla, traditional and simplified Chinese, Hindi, Khmer, Korean, Tagalog, Urdu, Vietnamese). Participants were recruited using a two-pronged approach: (1) community-based sampling that entailed direct outreach and in-person recruitment by partnering community organizations, and (2) population-based sampling using Qualtrics panels. The Association of Asian Pacific Community Health Organizations Institutional Review Board approved this study.

This study was conducted as part of a national effort commissioned by the Congressional Tri-Caucus and led by The Alliance of National Psychological Associations for Racial and Ethnic Equity and the National Urban League, in close partnership with community-based organizations, to gather data on the impacts of the COVID-19 pandemic on Black, Latinx, Asian American, NH/PI, American Indian, and Alaska Native populations (52).

### Measures

#### Financial concerns

Our main predictor variable was whether participants reported financial concerns as a source of stress from the COVID-19 pandemic (54). Participants were asked, “What have been your greatest sources of stress from the COVID-19 pandemic?” and were able to select up to 17 items (including financial concerns), “I am not stressed about the COVID-19 pandemic,” or “None of the above.” We define having financial concerns as a dichotomous variable, where participants were categorized as stressed or not stressed about financial concerns.

#### Psychological distress

The main outcome was psychological distress was measured using the Patient Health Questionnaire-4 (PHQ-4), that combines the PHQ-2 for depressive symptoms and Generalized Anxiety Disorder-2 (GAD-2) for anxiety symptoms. Participants were asked how often they were bothered over the last 7 days by “feeling nervous, anxious, or on edge,” “not being able to stop or control worrying,” “having little interest or pleasure in doing things,” and “feeling down, depressed, or hopeless.” The PHQ-4 is scored on a four-point Likert scale (0 = not at all, 1 = several days, 2 = more than half the days, 3 = nearly every day) (55). Psychological distress was analyzed as a continuous variable based on participant PHQ-4 scores ranging from 0 to 12. Past work has established the PHQ-4 as a validated screener for detecting anxiety and depressive disorders and a general marker for psychological distress (55, 56). For clinical utility and reporting the prevalence of this outcome, a PHQ-4 score ≥ 6 indicated psychological distress to dichotomize this variable (55, 56).

#### Benefit finding

Benefit finding was measured using the Perceived Benefits subscale from the COVID-19: Impact of the Pandemic and Health-Related Quality of Life in Cancer Patients and Survivors Questionnaire (57). Prior research has found internal consistency for this subscale as a protective factor to assess benefit finding in the context of the COVID-19 pandemic (58). Participants were asked about the extent to which they agreed or disagreed with statements about having a “greater appreciation for family and close friends,” “having a deeper appreciation for life,” “being more grateful for each day,” “being more accepting of things they cannot change,” and “finding new ways of connecting with family and friends,” since the breakout of the COVID-19 pandemic. Responses were reported on a five-point Likert scale ranging from −2 (strongly disagree) to 2 (strongly agree). We averaged scores from the five items to create a continuous measure of benefit finding from −2 to 2, where higher scores represent greater benefit finding (i.e., individuals expressed more appreciation and gratitude since the start of the COVID-19 pandemic). The Cronbach’s alpha value across the five items was 0.84, indicating an acceptable level of reliability and internal consistency of this scale (59).

#### Received pay

Received pay was analyzed as a binary variable from two questions. Participants were asked, “In the last 7 days, did you do ANY work for either pay or profit?” (yes or no) [Question 1]. If participants answered “no,” they were asked, “Are you receiving pay for the time you are not working?” [Question 2] This question included the following four options: (1) Yes, I use paid leave, (2) Yes, I receive full pay but do not have to take leave, (3) Yes, I receive partial pay, and (4) No, I receive no pay. If a participant responded with any of the first three options, then they were coded as having received pay. Thus, participants were categorized as having received pay if they responded “yes” to working for either pay or profit [Question 1] or answering “yes” to receiving pay for the time they are not working [Question 2].

#### Demographic characteristics

We accounted for possible confounding by sociodemographic characteristics, U.S. region of residence, survey language, and coping with stress through religious and spiritual practices. Asian race and ethnicity included seven Asian ethnic groups (Chinese [ref.], Filipino, Indian, Japanese, Korean, Pakistani, and Vietnamese), other Asian, multiethnic Asian, and multiracial Asian. The other Asian category included participants who self-identified as Bangladeshi, Cambodian, Indonesian, Malaysian, and Thai. Multiethnic Asian included participants who self-identified as two or more Asian ethnic groups and multiracial Asian included participants who self-identified as Asian race and another race (e.g., Black, White). Sociodemographic variables also included gender identity (man [ref.], woman, non-binary/trans/other), age (18–24 [ref.], 25–44, 45–64, 65 and over), nativity and years lived in the U.S. (U.S. born [ref.], foreign-born, lived in the U.S. for less than 10 years, lived in the U.S. for 10 years or more), marital status (single [ref.], married, divorced/separated/widowed), education level (less than high school graduate [ref.], high school graduate, some college or associate degree, bachelor’s degree, graduate degree), and annual household income (less than $25,000 [ref.], $25,000 to $34,999, $35,000 to $49,999, $50,000 to 74,999, $75,000 to $99,999, $100,000 or more). We also adjusted for U.S. region of residence (Northeast [ref.], Midwest, South, West) and survey language (English [ref.], Asian language).

Coping with stress through religious or spiritual practices was determined by the selecting “religious or spiritual practices (e.g., praying, reading religious texts)” to the question, “What have you done to cope with your stress related to the COVID-19 pandemic?” If respondents selected, “Religious or spiritual practices (e.g., praying, reading religious texts),” then they were coded as 1; all other respondents were coded as 0 or not using religious or spiritual practices for coping with stress for this variable. The relationship between religious coping and mental health among Asian Americans was explored during the context of the COVID-19 pandemic in relation to trauma through racism (60). Asian Americans were isolated from in-person religious social gatherings (60). Therefore, Asian American individuals who engaged in religious practices were deprived of the opportunity to build community through a religious capacity. For this reason, it is important to explore the relationship between religion and mental health among Asian American communities in the context of the COVID-19 pandemic in relation to financial concerns.

### Statistical analyses

Data weights for the Asian American sample were created using the 2019 American Community Survey (ACS) 1-year estimates and included Asian ethnicity, nativity, education, household income, gender, and age (53). We conducted weighted univariate analyses to describe demographic characteristics, financial concerns, benefit finding, received pay, and psychological distress for the overall sample. We used multivariable linear regression analyses to test for the associations between financial concerns and psychological distress (Model 1). We conducted complete case analysis for the regression models, which reduced 20.7% of analytic sample from 3,975 to 3,152 participants.

To examine whether benefit finding moderated the relationship between financial concerns and psychological distress, we included an interaction term for benefit finding with financial concerns (i.e., comparing people who reported financial concerns and low vs. high benefit finding scores) (Model 2). We also conducted a linear regression model to test the three-way interaction between financial concerns, benefit finding, and received pay on psychological distress (Model 3). These models were adjusted for sociodemographic characteristics, U.S. region of residence, survey language, and coping with stress through religious and spiritual practices. All analyses were conducted in Stata version 18.0, and statistical significance was designated as *p*-value < 0.05.

## Results

### Descriptive statistics

Table 1 includes weighted descriptive characteristics representing 22,371,683 Asian American adults in the U.S. from our sample of 3,152 respondents. Among our sample, 50.74% identified as a woman, and 42.80% were 25–44 years old. By Asian racial or ethnic group, from highest to lowest, 19.09% self-reported being Indian, 18.74% Chinese, 16.43% multiracial Asian, 13.75% Filipino, 9.53% other Asian ethnicity, 8.51% Vietnamese, 6.33% Korean, 3.35% Japanese, 2.20% multiethnic Asian, and 2.06% Pakistani. Most respondents were foreign-born (62.58%) and had lived in the U.S. for 10 or more years (50.54%), were married (59.90%), earned a bachelor’s degree or higher (67.19%), and had an annual household income of $100,000 or more (46.52%). Almost half of participants (48.88%) resided in the West, most participants completed the survey in English (85.49%), and 23.50% of participants reported coping with stress through religious or spiritual practices.

**Table 1:** Descriptive statistics for Asian American adults (*N* = 3,152).

Variable	Unweighted *N*	Weighted %
Gender identity		
Man	1,226	48.63
Woman	1,897	50.74
Non-binary, trans, other	29	0.63
Age group in years		
18–24	1,042	13.12
25–44	1,383	42.80
45–64	509	29.76
65+	218	14.32
Asian racial and ethnic group		
Chinese	699	18.74
Filipino	560	13.75
Indian	297	19.09
Japanese	53	3.35
Korean	412	6.33
Pakistani	68	2.06
Vietnamese	427	8.51
Other Asian ethnicity	137	9.53
Multiethnic Asian	253	2.20
Multiracial Asian	246	16.43
Nativity and years lived in the U.S.		
U.S. born	1,725	37.42
Foreign born, lived in U.S. <10 years	346	12.04
Foreign born, lived in U.S. 10 + years	1,081	50.54
Marital status		
Single	1,670	31.73
Married	1,315	59.90
Divorced, separated, or widowed	167	8.37
Education		
Less than high school graduate	93	8.87
High school graduate	371	9.60
Some college or associate degree	992	14.34
Bachelor’s degree	1,038	33.99
Graduate degree	658	33.20
Annual household income		
Less than $25,000	616	14.03
$25,000 to $34,999	259	4.63
$35,000 to $49,999	371	8.13
$50,000 to 74,999	502	14.18
$75,000 to $99,999	405	12.51
$100,000 or more	999	46.52
Survey language		
English	2,768	85.49
Asian language	384	14.51
U.S. region		
Northeast	380	12.95
Midwest	480	16.67
South	574	21.50
West	1,718	48.88
Coping		
Religious or spiritual practices	602	23.50

Source: 2021 Asian American and Native Hawaiian/Pacific Islander (AA & NH/PI) COVID-19 needs assessment study.

Table 2 shows the descriptive statistics for the main analytic variables: psychological distress, financial concerns, benefit finding, and received pay. Overall, Asian American adults reported an average psychological distress score of 3.40 (SD = 3.14), where scores ranged from 0 to 12, and higher scores represented higher levels of distress. 32.31% of Asian American adults reported psychological distress, 37.80% reported financial concerns, and 15.96% reported both psychological distress and financial concerns. Asian American participants reported an average benefit finding score of 0.96 (SD = 0.68), where scores ranged from −2 to 2, and higher scores represented higher benefit finding levels. Lastly, 71.50% of participants reported receiving pay (i.e., worked in the last 7 days or received pay for time not working).

**Table 2:** Main analytic variables for Asian American adults (*N* = 3,152).

Variable	Unweighted *N*	Weighted % or mean
Psychological distress (continuous, mean)*		3.40
Psychological distress (binary, %)	1,281	32.31
Financial concerns	1,369	37.80
Psychological distress and financial concerns	667	15.96
Benefit finding^†^		
Average score		0.96
I have greater appreciation for my family and close friends.		1.11
I have deeper appreciation for life.		1.05
I have been more grateful for each day.		0.91
I have been more accepting of things I cannot change.		0.82
I have found new ways of connecting with family and friends.		0.91
Received pay	2,090	71.50

Source: 2021 Asian American and Native Hawaiian/Pacific Islander (AA & NH/PI) COVID-19 needs assessment study.

^*^Psychological distress scores ranged from 0 to 12, where higher scores represent higher levels of distress.

† Benefit finding scores ranged from −2 to 2, where higher scores represent higher benefit finding (e.g., gratitude, appreciation).

### Financial concerns and psychological distress

Table 3 shows the beta coefficients and 95% confidence intervals (CIs) for the main effect model examining the relationship between financial concerns and psychological distress (Model 1), model testing the moderating effect of benefit finding on psychological distress (Model 2), and model testing the joint moderating effects of financial concerns, benefit finding, and received pay on psychological distress (Model 3). In the main effects model (Model 1), Asian American participants who reported financial concerns had a significant increase in psychological distress compared to those who did not report financial concerns (*β*: 1.24, 95% CI: 0.91, 1.56). For every one-unit increase in benefit finding score, there was a 0.96 decrease in reporting psychological distress (*β*: -0.96, 95% CI: −1.18, −0.73). In other words, Asian American participants who reported higher benefit finding levels were less likely to report psychological distress than participants who reported lower benefit finding levels, regardless of whether they reported financial concerns.

**Table 3:** Weighted linear regression predicting psychological distress for Asian American adults (*N* = 3,152).

Variable	Model 1		Model 2		Model 3	
	*β*	95% CI	*β*	95% CI	*β*	95% CI
Financial concerns	1.24	(0.91, 1.56)	1.57	(1.05, 2.09)	0.85	(−0.10, 1.79)
Benefit finding	−0.96	(−1.18, −0.73)	−0.82	(−1.11, −0.52)	−1.17	(−1.64, −0.70)
Received pay	0.16	(−0.19, 0.50)	0.16	(−0.18, 0.51)	−0.28	(−1.01, 0.45)
Interactions						
Financial concerns × benefit finding			−0.35	(−0.78, 0.07)	0.53	(−0.21, 1.27)
Financial concerns × received pay					1.03	(−0.07, 2.14)
Benefit finding × received pay					0.50	(−0.08, 1.07)
Financial concerns × benefit finding × received pay					−1.23	(−2.12, −0.34)
Gender identity						
Man						
Woman	0.63	(0.33, 0.92)	0.63	(0.33, 0.92)	0.62	(0.33, 0.92)
Non-binary, trans, other	2.20	(0.87, 3.52)	2.15	(0.80, 3.49)	2.16	(0.81, 3.51)
Age group in years						
18–24						
25–44	−0.45	(−0.87, −0.04)	−0.44	(−0.85, −0.02)	−0.43	(−0.84, −0.01)
45–64	−1.43	(−1.99, −0.88)	−1.42	(−1.98, −0.86)	−1.42	(−1.98, −0.87)
65+	−1.53	(−2.19, −0.87)	−1.51	(−2.17, −0.85)	−1.51	(−2.17, −0.86)
Asian racial and ethnic group						
Chinese						
Filipino	−0.14	(−0.51, 0.23)	−0.16	(−0.54, 0.21)	−0.18	(−0.56, 0.19)
Indian	−0.05	(−0.53, 0.43)	−0.06	(−0.54, 0.42)	−0.06	(−0.54, 0.42)
Japanese	0.08	(−0.99, 1.16)	0.09	(−0.98, 1.16)	0.07	(−0.97, 1.12)
Korean	−0.14	(−0.60, 0.32)	−0.14	(−0.60, 0.32)	−0.14	(−0.60, 0.32)
Pakistani	0.02	(−0.75, 0.79)	0.01	(−0.77, 0.78)	−0.01	(−0.79, 0.76)
Vietnamese	−0.23	(−0.71, 0.26)	−0.25	(−0.73, 0.24)	−0.25	(−0.73, 0.24)
Other Asian ethnicity	0.28	(−0.37, 0.92)	0.27	(−0.37, 0.92)	0.28	(−0.36, 0.93)
Multiethnic Asian	0.12	(−0.52, 0.76)	0.13	(−0.50, 0.77)	0.17	(−0.46, 0.80)
Multiracial Asian	0.26	(−0.28, 0.79)	0.23	(−0.30, 0.77)	0.23	(−0.30, 0.76)
Nativity and years lived in the U.S.						
U.S. born						
Foreign born, lived in U.S. <10 years	−0.65	(−1.11, −0.19)	−0.66	(−1.12, −0.20)	−0.66	(−1.11, −0.21)
Foreign born, lived in U.S. 10 + years	−0.52	(−0.86, −0.18)	−0.52	(−0.86, −0.18)	−0.51	(−0.86, −0.17)
Marital status						
Single						
Married	−0.40	(−0.78, −0.02)	−0.41	(−0.79, −0.04)	−0.41	(−0.79, −0.04)
Divorced, separated, or widowed	−0.20	(−0.87, 0.46)	−0.21	(−0.88, 0.45)	−0.23	(−0.88, 0.43)
Education						
Less than high school graduate	0.45	(−0.48, 1.37)	0.46	(−0.46, 1.39)	0.48	(−0.45, 1.41)
High school graduate	0.04	(−0.50, 0.58)	0.05	(−0.48, 0.59)	0.05	(−0.49, 0.58)
Some college or associate degree	0.37	(−0.04, 0.78)	0.38	(−0.03, 0.79)	0.39	(−0.02, 0.80)
Bachelor’s degree	0.06	(−0.29, 0.41)	0.06	(−0.28, 0.41)	0.07	(−0.27, 0.42)
Graduate degree						
Annual household income						
Less than $25,000	0.86	(0.41, 1.31)	0.85	(0.40, 1.30)	0.82	(0.37, 1.27)
$25,000 to $34,999	−0.10	(−0.64, 0.44)	−0.10	(−0.64, 0.44)	−0.09	(−0.64, 0.45)
$35,000 to $49,999	0.26	(−0.19, 0.71)	0.25	(−0.20, 0.70)	0.26	(−0.18, 0.70)
$50,000 to 74,999	0.12	(−0.32, 0.56)	0.12	(−0.32, 0.56)	0.13	(−0.31, 0.57)
$75,000 to $99,999	0.47	(0.01, 0.93)	0.48	(0.02, 0.94)	0.48	(0.02, 0.94)
$100,000 or more						
Survey language						
English						
Asian language	0.24	(−0.24, 0.72)	0.25	(−0.23, 0.73)	0.26	(−0.21, 0.73)
U.S. Region						
Northeast	−0.69	(−1.10, −0.28)	−0.71	(−1.12, −0.30)	−0.73	(−1.14, −0.33)
Midwest	−0.19	(−0.62, 0.25)	−0.21	(−0.65, 0.23)	−0.22	(−0.66, 0.21)
South	−0.34	(−0.72, 0.04)	−0.34	(−0.72, 0.04)	−0.35	(−0.73, 0.03)
West						
Coping with religious or spiritual practices	0.56	(0.19, 0.92)	0.57	(0.20, 0.93)	0.59	(0.23, 0.95)
*R* ^2^	0.23		0.23		0.24	

Source: 2021 Asian American and Native Hawaiian/Pacific Islander (AA & NH/PI) COVID-19 needs assessment study.

### Two-way interaction of financial concerns x benefit finding on psychological distress

In the model testing the moderating effect of benefit finding on financial concerns and psychological distress (Model 2), results from the main effects model (Model 1) remained statistically significant. Participants who reported financial concerns had a significant increase in psychological distress than those who did not report financial concerns (*β*: 1.57, 95% CI: 1.05, 2.09). Participants with higher benefit finding levels had significantly lower levels of psychological distress (*β*: -0.82, 95% CI: −1.11, −0.52). Benefit finding did not moderate the relationship between financial concerns and psychological distress (β: -0.35, 95% CI: −0.78, 0.07). In other words, there was no difference in the relationship between financial concerns and psychological distress by benefit finding levels among Asian American participants.

### Three-way interaction of financial concerns x benefit finding x received pay on psychological distress

**Figure 1:**
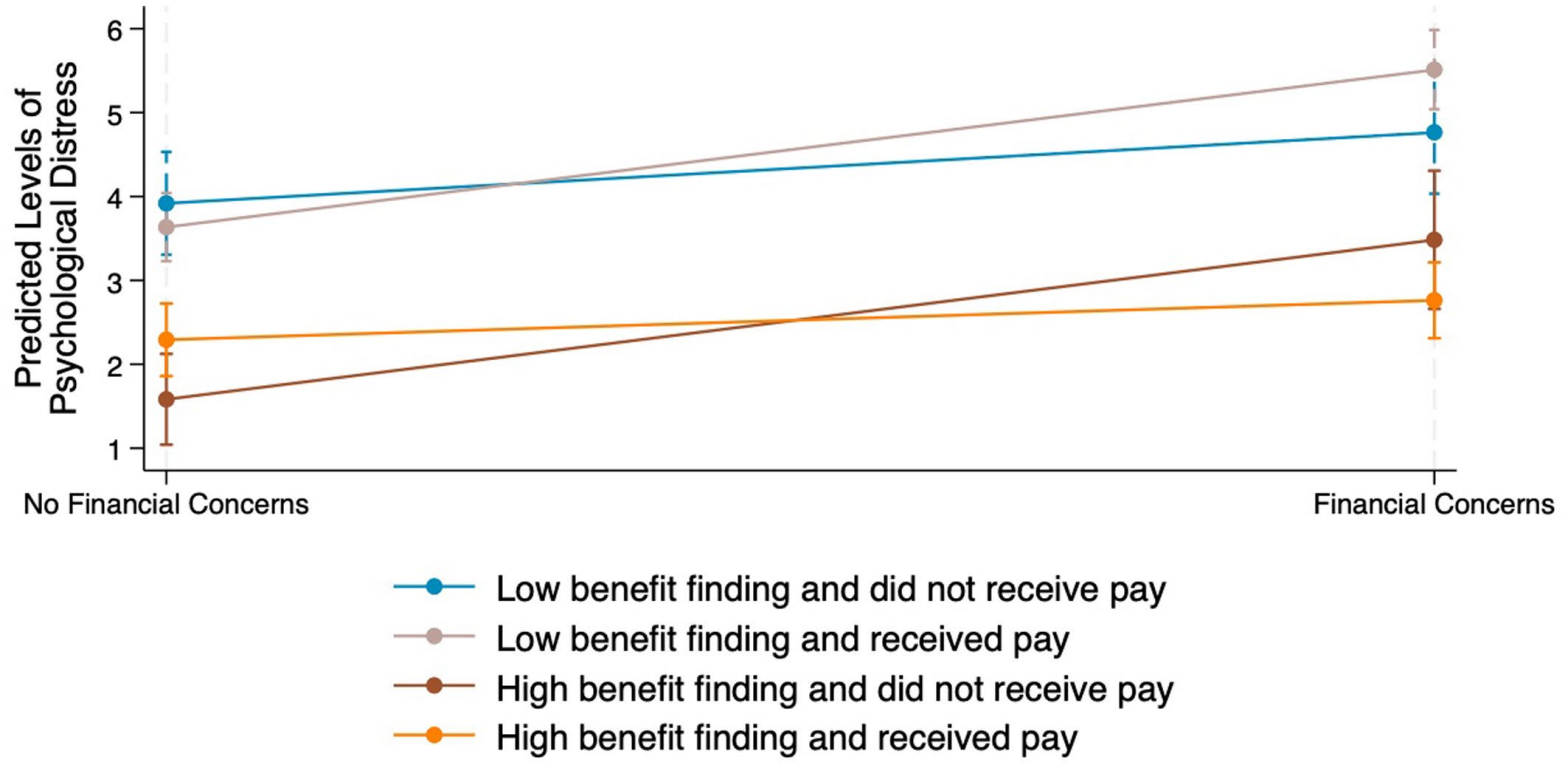
Weighted linear regression predicted levels of psychological distress by financial concerns, benefit finding, and received pay. Source: 2021 Asian American and Native Hawaiian/Pacific Islander (AA & NH/PI) COVID-19 Needs Assessment Study.

Lastly, we tested the joint effects of financial concerns, benefit finding, and received pay on psychological distress (Model 3). Like the prior models, higher benefit finding scores were significantly associated with lower psychological distress (*β*: -1.17, 95% CI: −1.64, −0.70); there were no statistical differences in psychological distress among participants because of financial concerns or received pay. The three-way interaction term between financial concerns, benefit finding, and received pay significantly modified psychological distress (β: -1.23, 95% CI: −2.12, −0.34); the level of psychological distress was reduced by 1.23 for participants when considering these three variables. Figure 1 visually shows the varying predicted levels of psychological distress; higher benefit finding and receiving pay was the most protective combination against psychological distress among participants with financial distress.

In summary, Asian American participants who reported financial concerns were more likely to experience psychological distress than those with no financial concerns, and participants with greater benefit finding levels were less likely to experience psychological distress than those with lower benefit finding levels. Additionally, while benefit finding alone did not change the relationship between financial concerns and psychological distress, the combination of benefit finding and received pay significantly moderated this relationship.

## Discussion

In this study, our key finding was that among those with financial concerns, participants who reported higher levels of benefit finding *and* received pay had lower levels of experiencing psychological distress, as compared to those who only reported high benefit-finding scores. More than a third (37.8%) of participants reported that financial concerns were a major source of stress from the COVID-19 pandemic. These findings are important to inform our understanding of Asian Americans’ coping with financial hardships, as well as enact individual- and policy-level interventions that provide economic assistance, when considering the significant and disproportionate financial insecurity that exacerbated and persisted in Asian Americans with limited English proficiency during the COVID-19 pandemic (61).

### Benefit finding and financial concerns

Based on our results, we find that our original hypothesis was not fully supported, where benefit finding alone as a meaning-based coping mechanism was not enough to significantly change the appraisal process of financial concerns and psychological distress. Therefore, before accounting for factors like received pay, financial concerns and benefit finding had independent roles to shape psychological distress in Asian Americans. However, higher benefit finding was associated with lower psychological distress among Asian Americans independent of COVID-19 related financial concerns. This finding is aligned with the early pandemic research suggesting an association between benefit finding in the beginning of the pandemic and positive outcomes (42–44). However, protective factors such as benefit finding may vary by stressor types, and the evidence of benefit finding buffering against financial stress appears to be mixed in previous literature (62). In another study, benefit finding did not moderate the associations between financial stress and depressive symptoms in mostly non-Hispanic White high school and university students in COVID-19 (62). Instead, the researchers found that benefit finding was associated with lower depressive symptoms, regardless of differences in financial stress levels for both samples.

The differences between our study and Scott et al. (33) could potentially be explained by the benefit finding measure used. Scott et al. (62) used a one-item self-report question from the COVID-19 Adolescent Symptom and Psychological Experience Questionnaire that asks participants to select from a list of benefits they perceived during the COVID-19 pandemic (e.g., reduced amount of schoolwork, getting more sleep, spending more time with family). In our study, we adapted a five-item perceived benefits scale from the COVID-19 Impact Survey that was originally used among individuals with cancer (57). Scale items included having a “greater appreciation for family and close friends” and “having a deeper appreciation for life.” The two benefit finding measures appeared quite different in terms of the factor of benefit finding being measured, which is another challenge that contributes to the inconsistent relations between benefit finding and health outcomes (33).

Another reason that contributes to variable findings may be the racial and ethnic composition of the study samples. A meta-analysis of 87 benefit finding cross-sectional studies found that benefit finding was more strongly associated with better health outcomes when minoritized racial and ethnic respondents comprised 25% or more of the sample (33). It is unclear why this might be the case and whether there could be cultural differences in benefit finding. To the best of our knowledge, this is the first study that examined benefit finding and psychological distress among Asian Americans in the pandemic context of financial concerns. Future research should explore how Asian Americans interpret and experience benefit finding and examine the mechanisms in which benefit finding may help buffer against the psychological effects of financial stress among Asian Americans.

### Benefit finding and received pay

Notably, our study measured both benefit finding (a cognitive-based coping strategy) and received pay (as a form of financial support) to understand their role in potentially reducing financial stress during the pandemic. We found that the presence of both benefit finding and received pay protects Asian American participants the most from experiencing psychological distress when they have financial concerns. This is particularly important as Asian Americans’ employment was most negatively affected by the COVID-19 lockdown than other racial groups, with individuals with lower education attainment more likely to lose work and less likely to regain employment in the reopening period (15). Our finding suggests that in times of financial concerns specific to the COVID-19 pandemic, which was a proximal and uncontrollable stressor, Asian Americans benefitted the most from employing cognitive-based reappraisal strategies like benefit finding and having concrete financial help such as receiving pay for work or for the time not at work that support their basic needs. It is likely that when stress is especially high and individuals’ livelihoods are threatened, it becomes harder for people to engage with cognitive-based skills like benefit finding to cope with stress. A pre-pandemic study conducted with survivors of cancer found that the combination of financial difficulty and worry about affording care was negatively associated with making positive changes, which is a crucial aspect of benefit finding (63). For Asian Americans who experienced increased discrimination and co-occurring financial and health challenges during the pandemic, benefit finding and receiving pay might be equally important to them in navigating individual- and structural-level stressors (64, 65). To ensure economic recovery from the pandemic for vulnerable Asian American subpopulations (e.g., groups with low education attainment), not only will we need culturally responsive interventions that promote benefit finding coping strategies, but we will also need more effective policies to comprehensively improve economic security, such as expanding Supplemental Nutrition Assistance Program (SNAP) benefits, increasing enrollment of eligible individuals into federal assistance programs, and expanding eligibility criteria for new immigrants.

Because our results show a buffering effect between financial concerns and psychological distress only when benefit finding and received pay are both present, it is likely that received pay may be a means to better enable individuals to engage in benefit finding. This can be better understood by the conservation of resources (COR) theory, where resource gains hold importance in the face of stressors beyond an individual’s personal appraisal process (66). In addition to appraisal-based theories, COR is a resource-based theory that considers environmental contexts, resources, and internal cognitive processes as relevant components of the stress process (66). Particularly during the COVID-19 pandemic, Asian Americans were prone to facing multiple stressors (e.g., racism, COVID-19 infection), and COR posits that in times of stress, people focus on objective circumstances first before their own perceptions or stress appraisal (66). This suggests that perhaps when facing financial concerns, Asian Americans will likely gain greater mental health benefits from receiving instrumental means of support (i.e., received pay) and cognitive reappraisal. Thus, our work supports the notion that the interaction of both structural and personal resources matters most for mental health, rather than benefit finding or receiving pay alone, during periods of acute financial stress for Asian Americans.

Based on our findings, there is great promise to better understand the threshold at which benefit finding becomes feasible after receiving a certain level of structural support addressing basic needs. While this threshold question is beyond the scope of the current study, which takes place during COVID-19 specifically, future research directions could examine interactions between individual-level coping strategies and varying levels of receiving community resources. This invites other researchers to move beyond the transactional model of stress and coping and consider how COR can help inform the need to enhance both psychological resilience and policy-level interventions to meet basic needs. Especially for populations that face unmet basic needs such as food, housing, and job insecurity, it will be critical to know when meaning-focused coping strategies and positive reappraisal become effective after receiving structural support to protect against mental health consequences.

### Study strengths

There are notable strengths in our study. First, this study analyzed a large sample of Asian Americans with several ethnic subgroups. Secondly, data was collected during a heightened, critical period, between January and April 2021, in the COVID-19 pandemic. With the rise in anti-Asian hate and abrupt lifestyle changes, this was a crucial period revealing the detrimental impacts of pandemic stressors on well-being. Lastly, our research is one of the first studies to examine benefit finding among Asian Americans who report COVID-19-related financial concerns. This survey was co-developed and administered in close collaboration with community-based organizations and their respective community efforts, as well as a survey research panel, which provides a robust and diverse national sample of Asian American adults who are often not included in health survey research.

### Study limitations

There are several limitations in our study. First, we used one measure of benefit finding, which was an average of five items, that may provide less sensitivity for this construct to be contextualized. This benefit finding scale has also not been validated in Asian American populations. Future research should test the psychometric properties of this scale and test adapted or translated versions for specific Asian ethnic groups or subpopulations. Second, while disaggregated ethnicity data were available, we did not examine the differential role of benefit finding for specific Asian ethnic groups because we hypothesized that benefit finding profiles and experiences would be similar across our Asian American sample. However, we acknowledge a longstanding pattern in research where Asian Americans are treated as a uniform group, despite literature citing their subgroup diversity (67). Such aggregation may mask the substantial heterogeneity among Asians, including migration histories, socioeconomic status, culture, exposure to stressors, and access to resources that shape the lived experiences of individuals (67). For example, prior work reveals that financial strain varies across Asian subgroups, with Southeast Asian communities being more likely to experience economic hardship and subsequent mental health outcomes (24). Thus, future research directions may include disaggregating data or examining specific Asian subgroups to better capture these differences and potentially identify unique mechanisms that can inform targeted psychosocial and public health interventions. Lastly, our data were cross-sectional, and we could not estimate causal relationships between financial stress, benefit finding, and psychological distress. Due to our analytic variables being measured at the same time point in this cross-sectional survey, there is the possibility for reverse causality. For instance, individuals with higher levels of psychological distress may subsequently be more likely to perceive stress from financial concerns or report lower levels of benefit finding.

### Future directions

#### Psychology-based implications

This study highlights the protective role of benefit finding against psychological distress for the Asian American community. Although we focused on coping with financial concerns during the COVID-19 pandemic, benefit finding could be learned and strengthened among individuals for more positive coping responses to other stressors experienced and during this post-pandemic period. Mental health professionals could develop targeted interventions that incorporate benefit finding as a positive coping strategy to promote well-being.

Furthermore, psychological interventions should consider coping strategies and potential effectiveness in the context in which the experience occurs, including social and cultural worldviews and values (68). For example, older generations, who may have been predominantly raised in a collectivistic context, are more aligned with their collectivistic culture’s values and traditions; therefore, culturally tailored interventions may more strongly benefit older-generation Asians (69, 70). We found that the combination of benefit finding and received pay attenuated the effect of financial concerns on psychological distress among Asian Americans; thus, it is essential for psychological interventions to consider tangible forms of support in addition to meaning-based coping mechanisms when assisting clients facing key stressors. Additionally, mental health professionals and researchers are encouraged to examine other forms of coping and stressors beyond the context of the COVID-19 pandemic. To develop our understanding of benefit finding in this population further, future research should explore how Asian Americans articulate and experience benefits in the context of financial and racialized stress. Qualitative and mixed-methods approaches may be especially valuable in capturing the nuanced and culturally specific ways this community derives meaning from adverse life events (71, 72). Lastly, it is crucial for mental health practitioners to build greater awareness and workforce capacity to understand and address the breadth of mental health experiences for minoritized communities. Practitioners must identify and promote clients’ existing strengths and positive coping strategies and foster a strong sense of racial, ethnic and cultural identity – to expand the boundaries and innovate towards what a culturally responsive and relevant intervention could entail.

#### Public health policy implications

While this study focused on psychological distress and financial concerns during the COVID-19 pandemic, Asian American people also experienced anti-Asian discrimination and hate crimes that also compounded and worsened their health and mental health (65). The increase in Asian American hate crimes, especially during the COVID-19 pandemic, resulted in President Joseph Biden signing the COVID-19 Hate Crimes Act into law in May 2021 (73) and both state and local-level efforts, like the establishment of hate crime task forces such as the New York Department Asian Hate Crime Task Force, California’s Racial Justice Bureau, and Asian Americans United Against Violence (74). These legislative measures and initiatives combat hate crimes that target Asian American communities, yet more holistic and multisector action is needed to actively address and support Asian Americans’ mental health and well-being, particularly promoting the role of benefit finding in mitigating the adverse effects of Asian American-specific experiences, such as economic stressors amid anti-Asian racism during the COVID-19 pandemic.

Moreover, this study reinforces the need for economic policies, such as paid sick leave, expanded unemployment insurance, employment protections, stimulus payments, and equitable allocation of government funding, to be recognized as public health interventions that may reduce financial strain and strengthen mental well-being (75, 76). Furthermore, this underscores an urgent call for integrated policy approaches that pair economic support with proactive coping tools to enhance population mental health, especially for communities navigating both economic and race-based stressors. Specifically, future policy initiatives may integrate benefit finding to promote posttraumatic growth following stressful life events, as it has been shown to enhance overall health and mood due to increased expressions of gratitude, decreased distress levels, and reduced anxiety levels (44, 45). For example, government funding may be leveraged to support Asian American-serving community-based organizations in delivering benefit finding-based workshops alongside financial aid, thereby strengthening community cohesion, social support, and individual belonging to impact health positively (77). As such, these efforts may create opportunities to shift attention toward meaning-making and growth among impacted individuals, rather than focusing solely on the negative effects of distress-inducing circumstances and life events (33). In this way, public health efforts not only encourage adaptive coping but also advocate for social and economic policies that create the conditions in which such coping can meaningfully occur. Lastly, while the present study focused on the unique experiences of the Asian American community during the COVID-19 pandemic, these recommendations present an opportunity to initiate and foster collective community action in allyship with other impacted communities to promote social justice and health equity (77).

## Conclusion

In our study, Asian Americans with greater benefit finding had lower psychological distress, and benefit finding moderated the relationship between financial concerns and psychological distress. We encourage mental health and allied health professionals to build and strengthen positive psychological responses, like benefit finding, as potential coping mechanisms to mitigate economic or other stress for diverse Asian American communities. Alongside this, structural-level solutions like equitable allocation of government funding and holistic health across all legislative action are needed to alleviate the co-occurring stressors impacting minoritized racial and ethnic communities. Future research should explore the relationships between benefit finding and other health outcomes over the life course (e.g., different disease stages), as well as additional other protective coping responses for Asian Americans as a racial group and potential differences by Asian ethnicity or specific subgroups. Considering the increasing rates of adverse mental health outcomes among Asian American communities, it is essential to integrate meaning-based coping strategies with equitable and tangible resources to address the needs of this population.

## Data Availability

The data analyzed in this study is subject to the following licenses/restrictions: De-identified data and materials are available upon reasonable request. Requests to access these datasets should be directed to AS, asaw@depaul.edu.

## References

[1] Clawson AH Nwankwo CN Blair AL Pepper-Davis M Ruppe NM Cole AB. COVID-19 impacts on families of color and families of children with asthma. J Pediatr Psychol. (2021) 46:378–91. doi: 10.1093/jpepsy/jsab021, 33738483 PMC7989447

[2] Karpman M Gonzalez D Kenney GM Parents are struggling to provide for their families during the pandemic: material hardships greatest among low-income, black, and Hispanic parents. Urban Institute (2020).

[3] Sangalang CC. “I’m sick of being called a hero – I want to get paid like one”: Filipino American frontline workers’ health under conditions of COVID-19 and racial capitalism. Front Public Health. (2022) 10:977955. doi: 10.3389/fpubh.2022.977955, 36504981 PMC9726904

[4] Yan BW Hwang AL Ng F Chu JN Tsoh JY Nguyen TT. Death toll of COVID-19 on Asian Americans: disparities revealed. J Gen Intern Med. (2021) 36:3545–9. doi: 10.47602/jpsp.v5i2.245, 34347256 PMC8335981

[5] Goldman N Pebley AR Lee K Andrasfay T Pratt B. Racial and ethnic differentials in COVID-19-related job exposures by occupational standing in the US. PLoS One. (2021) 16:e0256085. doi: 10.1371/journal.pone.0256085, 34469440 PMC8409606

[6] Wang D Gee GC Bahiru E Yang EH Hsu JJ. Asian-Americans and Pacific islanders in COVID-19: emerging disparities amid discrimination. J Gen Intern Med. (2020) 35:3685–8. doi: 10.1007/s11606-020-06264-5, 33009656 PMC7531609

[7] Kalyanaraman Marcello R Dolle J Tariq A Kaur S Wong L Curcio J . Disaggregating Asian race reveals COVID-19 disparities among Asian American patients at new York City’s public hospital system. Public Health Rep. (2022) 137:317–25. doi: 10.1177/00333549211061313, 34965776 PMC8900247

[8] Lee J Ramakrishnan K Wong J. Accurately counting Asian Americans is a civil rights issue. Ann Am Acad Polit Soc Sci. (2018) 677:191–202. doi: 10.1177/0002716218765432

[9] Le TK Cha L Han H-R Tseng W. Anti-Asian xenophobia and Asian American COVID-19 disparities. Am J Public Health. (2020) 110:1371–3. doi: 10.2105/AJPH.2020.305846, 32783714 PMC7427240

[10] Chen L Young M-EDT Rodriguez MA Kietzman K. Immigrants’ enforcement experiences and concern about accessing public benefits or services. J Immigr Minor Health. (2023) 25:1077–84. doi: 10.1007/s10903-023-01460-x, 36859637 PMC10509127

[11] Ma KPK Bacong AM Kwon SC Yi SS Ðoàn LN. The impact of structural inequities on older Asian Americans during COVID-19. Front Public Health. (2021) 9:690014. doi: 10.3389/fpubh.2021.690014, 34490181 PMC8417937

[12] Quach T Ðoàn LN Liou J Ponce NA. A rapid assessment of the impact of COVID-19 on Asian Americans: cross-sectional survey study. JMIR Public Health Surveill. (2021) 7:e23976. doi: 10.2196/23976, 34019478 PMC8202653

[13] Yi SS Ali SH Russo RG Foster V Radee A Chong S . COVID-19 leads to dramatic changes in the food retail environment in new York City: May-July 2020. J Immigr Minor Health. (2022) 24:31–7. doi: 10.1007/s10903-021-01230-7, 34258716 PMC8277094

[14] Bartik AW Bertrand M Lin F Rothstein J Unrath M. Measuring the Labor Market at the Onset of the COVID-19 Crisis. Cambridge: National Bureau of Economic Research (2020).

[15] Kim AT Kim C Tuttle SE Zhang Y. COVID-19 and the decline in Asian American employment. Res Soc Stratific Mobil. (2021) 71:100563. doi: 10.1016/j.rssm.2020.100563, 33052161 PMC7543758

[16] Gemelas J Davison J Keltner C Ing S. Inequities in employment by race, ethnicity, and sector during COVID-19. J Racial Ethnic Health Disparities. (2022) 9:350–5. doi: 10.1007/s40615-021-00963-3, 33452573 PMC7810107

[17] McGarity-Palmer R Saw A Tsoh JY Yellow Horse AJ. Trends in racial discrimination experiences for Asian Americans during the COVID-19 pandemic. J Racial Ethnic Health Disparities. (2024) 11:168–83. doi: 10.1007/s40615-022-01508-y, 36602751 PMC9815050

[18] Gover AR Harper SB Langton L. Anti-Asian hate crime during the COVID-19 pandemic: exploring the reproduction of inequality. Am J Crim Just. (2020) 45:647–67. doi: 10.1007/s12103-020-09545-1, 32837171 PMC7364747

[19] Jeung R Horse AY Popovic T Lim R. Stop AAPI hate national report. Ethnic Stud Rev. (2021) 44:19–26. doi: 10.1525/esr.2021.44.2.19

[20] Tessler H Choi M Kao G. The anxiety of being Asian American: hate crimes and negative biases during the COVID-19 pandemic. Am J Crim Just. (2020) 45:636–46. doi: 10.1007/s12103-020-09541-5, 32837158 PMC7286555

[21] Lozano P Rueger SY Lam H Louie N Southworth A Maene C . Prevalence of depression symptoms before and during the COVID-19 pandemic among two Asian American ethnic groups. J Immigrant Minority Health. (2022) 24:909–17. doi: 10.1007/s10903-021-01287-4, 34643848 PMC8511614

[22] Zhou S Banawa R Oh H The mental health impact of COVID-19 racial and ethnic discrimination against Asian American and Pacific islanders Front Psych 2021 12:708426 doi: 10.3389/fpsyt.2021.708426 34867510 PMC8637907

[23] Matthews TA Chen L Chen Z Han X Shi L Li Y . Negative employment changes during the COVID-19 pandemic and psychological distress: evidence from a nationally representative survey in the U.S. J Occup Environ Med. (2021) 63:931–7. doi: 10.1097/JOM.0000000000002325, 34267107 PMC8562921

[24] Islam JY Awan I Kapadia F. Food insecurity, financial hardship, and mental health among multiple Asian American ethnic groups: findings from the 2020 COVID-19 household impact survey. Health Equity. (2022) 6:435–47. doi: 10.1089/heq.2021.0179, 35801150 PMC9257551

[25] Lee J Howard JT. Increased self-reported mental health problems among Asian-Americans during the COVID-19 pandemic in the United States: evidence from a nationally representative database. J Racial Ethnic Health Disparities. (2023) 10:2344–53. doi: 10.1007/s40615-022-01414-3, 36129608 PMC9491255

[26] Tiwari BB Zhang D “Stacy.” Differences in mental health status among Asian Americans during the COVID-19 pandemic: findings from the health, ethnicity, and pandemic study. Health Equity (2022) 6:448–453. doi: 10.1089/heq.2022.0029, 35801151 PMC9257548

[27] Berjot S Gillet N. Stress and coping with discrimination and stigmatization. Front Psychol. (2011) 2:33. doi: 10.3389/fpsyg.2011.00033, 21713247 PMC3110961

[28] Lazarus RS Folkman S. Stress, Appraisal, and Coping. New York: Springer Publishing Company (1984).

[29] Faryabi R Rahimi T Daneshi S Movahed E Yusefi AR Shahrokhabadi MS . Stress coping styles in family and relatives of coronavirus disease 2019 (COVID-19) patients in the south of Iran: application of Lazarus and Folkman’s theory of stress coping. Open Public Health J. (2022) 6:1–8. doi: 10.2174/18749445-v15-e220927-2021-243

[30] Huynh MP Yellow Horse AJ Mai NM Mantuhac J Saw A. Discrimination and psychological distress among Asian Americans during COVID-19: gender differences in the moderating role of social support. Am J Orthopsychiatry. (2024) 94:23–32. doi: 10.1037/ort0000702, 37768606

[31] Shakespeare-Finch J Bowen-Salter H Cashin M Badawi A Wells R Rosenbaum S . COVID-19: an Australian perspective. J Loss Trauma. (2020) 25:662–72. doi: 10.1080/15325024.2020.1780748

[32] Tedeschi RG Calhoun LG. The posttraumatic growth inventory: measuring the positive legacy of trauma. J Trauma Stress. (1996) 9:455–71. doi: 10.1007/BF02103658, 8827649

[33] Helgeson VS Reynolds KA Tomich PL. A meta-analytic review of benefit finding and growth. J Consult Clin Psychol. (2006) 74:797–816. doi: 10.1037/0022-006X.74.5.797, 17032085

[34] Cassidy T Giles M McLaughlin M. Benefit finding and resilience in child caregivers. Br J Health Psychol. (2014) 19:606–18. doi: 10.1111/bjhp.12059, 23869796

[35] Liu Z Thong MSY Doege D Koch-Gallenkamp L Bertram H Eberle A . Prevalence of benefit finding and posttraumatic growth in long-term cancer survivors: results from a multi-regional population-based survey in Germany. Br J Cancer. (2021) 125:877–83. doi: 10.1038/s41416-021-01473-z, 34215852 PMC8437934

[36] Ma L Zhu K Shi C Chen X Gao Y Cai C . Association between the patients’ symptom burden and their family caregivers’ benefit finding in non-small cell lung cancer receiving combined chemotherapy. Support Care Cancer. (2023) 31:148. doi: 10.1007/s00520-023-07590-0, 36729306

[37] Holland JM Currier JM Neimeyer RA. Meaning reconstruction in the first two years of bereavement: the role of sense-making and benefit-finding. Omega (Westport). (2006) 53:175–91. doi: 10.2190/FKM2-YJTY-F9VV-9XWY

[38] Sears SR Stanton AL Danoff-Burg S. The yellow brick road and the emerald city: benefit finding, positive reappraisal coping and posttraumatic growth in women with early-stage breast cancer. Health Psychol. (2003) 22:487–97. doi: 10.1037/0278-6133.22.5.487, 14570532

[39] Folkman S. The case for positive emotions in the stress process. Anxiety Stress Coping. (2008) 21:3–14. doi: 10.1080/10615800701740457, 18027121

[40] Lee S Juon H-S Martinez G Hsu CE Robinson ES Bawa J . Model minority at risk: expressed needs of mental health by Asian American young adults. J Community Health. (2008) 34:144–52. doi: 10.1007/s10900-008-9137-1, 18931893 PMC3296234

[41] Lim AJ Lau CYH Cheng C-Y. Applying the dual filial piety model in the United States: a comparison of filial piety between Asian Americans and Caucasian Americans. Front Psychol. (2022) 12:786609. doi: 10.3389/fpsyg.2021.786609, 35185688 PMC8850268

[42] Cox CR Swets JA Gully B Xiao J Yraguen M. Death concerns, benefit-finding, and well-being during the COVID-19 pandemic. Front Psychol. (2021) 12:648609. doi: 10.3389/fpsyg.2021.648609, 34093336 PMC8170023

[43] Tang S Xiang M Cheung T Xiang Y-T. Mental health and its correlates among children and adolescents during COVID-19 school closure: the importance of parent-child discussion. J Affect Disord. (2021) 279:353–60. doi: 10.1016/j.jad.2020.10.016, 33099049 PMC7550131

[44] August R Dapkewicz A. Benefit finding in the COVID-19 pandemic: college students’ positive coping strategies. J Posit Sch Psychol. (2021) 5:73–86. doi: 10.47602/jpsp.v5i2.245

[45] Hansen SR Wetherell MA Smith MA. Written benefit finding for improving psychological health during the Covid-19 pandemic first wave lockdown. Psychol Health. (2022) 37:1223–40. doi: 10.1080/08870446.2021.1936521, 34130556

[46] Moore JB Rubin KCR Heaney CA. Benefit finding and well-being over the course of the COVID-19 pandemic. PLoS One. (2023) 18:e0288332. doi: 10.1371/journal.pone.0288332, 37498840 PMC10374125

[47] Gonzalez P Nuñez A Wang-Letzkus M Lim J-W Flores KF Nápoles AM. Coping with breast cancer: reflections from Chinese American, Korean American, and Mexican American women. Health Psychol. (2016) 35:19–28. doi: 10.1037/hea0000263, 26389720 PMC4695243

[48] Garrison ST Rampold SD Vasquez K Gillen M Baker LM. Parents’ employment, income, and finances before and during the COVID-19 pandemic. J Consum Aff. (2022) 56:276–91. doi: 10.1111/joca.12443, 35603323 PMC9115126

[49] Kowalski RM Carroll H Britt J. Finding the silver lining in the COVID-19 crisis. J Health Psychol. (2022) 27:1507–14. doi: 10.1177/1359105321999088, 33645297

[50] Couch KA Fairlie RW Xu H. Early evidence of the impacts of COVID-19 on minority unemployment. J Public Econ. (2020) 192:104287. doi: 10.1016/j.jpubeco.2020.104287, 32952224 PMC7489888

[51] Langford CPH Bowsher J Maloney JP Lillis PP. Social support: a conceptual analysis. J Adv Nurs. (1997) 25:95–100. doi: 10.1046/j.1365-2648.1997.1997025095.x, 9004016

[52] Grills C Carlos Chavez FL Saw A Walters KL Burlew K Randolph Cunningham SM . Applying culturalist methodologies to discern COVID-19’s impact on communities of color. J Community Psychol. (2022) 51:2331–54. doi: 10.1002/jcop.22802, 35102549 PMC9015500

[53] McGarity-Palmer R Saw A Sun M Huynh MP Takeuchi D. Mental health needs among Asian and Asian American adults during the COVID-19 pandemic. Public Health Rep. (2023) 138:535–45. doi: 10.1177/00333549231156566, 36971268 PMC10051010

[54] Nozadi SS Li X Kong X Rennie B Kanda D MacKenzie D . Effects of COVID-19 financial and social hardships on infants’ and toddlers’ development in the ECHO program. Int J Environ Res Public Health. (2023) 20:1013. doi: 10.3390/ijerph20021013, 36673770 PMC9858743

[55] Kroenke K Spitzer RL Williams JBW Löwe B. An ultra-brief screening scale for anxiety and depression: the PHQ–4. Psychosomatics. (2009) 50:613–21. doi: 10.1016/S0033-3182(09)70864-3, 19996233

[56] Löwe B Wahl I Rose M Spitzer C Glaesmer H Wingenfeld K . A 4-item measure of depression and anxiety: validation and standardization of the patient health Questionnaire-4 (PHQ-4) in the general population. J Affect Disord. (2010) 122:86–95. doi: 10.1016/j.jad.2009.06.019, 19616305

[57] Penedo FJ Cohen L Bower J Antoni MH COVID-19: impact of the pandemic and HRQOL in cancer patients and survivors. Measure Validation Scale. (2020).

[58] Saez-Clarke E Otto AK Prinsloo S Natori A Wagner RW Gomez TI . Development and initial psychometric evaluation of a COVID-related psychosocial experiences questionnaire for cancer survivors. Qual Life Res. (2023) 32:3475–94. doi: 10.1007/s11136-023-03456-4, 37358738 PMC11817160

[59] Heale R Twycross A. Validity and reliability in quantitative studies. Evid Based Nurs. (2015) 18:66–7. doi: 10.1136/eb-2015-102129, 25979629

[60] Nie F. Asian hate, minority stress, and religious coping: a study of Asian and Asian American adults in the USA during the COVID-19 pandemic. J Relig Health. (2023) 62:681–701. doi: 10.1007/s10943-022-01693-4, 36394690 PMC9669543

[61] Lu I Suss R Lanza DV Cohen S Yusuf Y Yi SS. A qualitative study to inform the development of a subsidized community-supported agriculture program for Chinese Americans in Brooklyn, New York, U.S. Prev Med Rep. (2023) 36:102480. doi: 10.1016/j.pmedr.2023.102480, 37920594 PMC10618813

[62] Scott SR Rozek CS Wolfe GR Fox KR Doom JR. Finding silver linings: benefit-finding, stress, and depressive symptoms during the COVID-19 pandemic. ADV RES SCI. (2024) 6:95–103. doi: 10.1007/s42844-024-00147-y, 41431717

[63] Jones SMW Walker R Fujii M Nekhlyudov L Rabin BA Chubak J. Financial difficulty, worry about affording care, and benefit finding in long-term survivors of cancer. Psycho-Oncology. (2018) 27:1320–6. doi: 10.1002/pon.4677, 29462511 PMC13088915

[64] McGarity-Palmer R Saw A Horse AJY Yi SS Tsoh J Takeuchi D. Profiles of a COVID-19 syndemic: anti-Asian racism, economic challenges, and mental and physical health. J Racial Ethnic Health Disparities. (2024) 11:300–12. doi: 10.1007/s40615-023-01519-3, 36692660 PMC9872729

[65] Saw A Yi SS Ðoàn LN Tsoh JY Horse AJY Kwon SC . Improving Asian American health during the syndemic of COVID-19 and racism. E Clin Med. (2022) 45:101313. doi: 10.1016/j.eclinm.2022.101313, 35233516 PMC8881903

[66] Hobfoll SE. The influence of culture, community, and the nested-self in the stress process: advancing conservation of resources theory. Appl Psychol. (2001) 50:337–421. doi: 10.1111/1464-0597.00062

[67] Sue S Sue D Sue DW. Who are the Asian Americans? Commentary on the Asian American psychology special issue. Am Psychol. (2021) 76:689–92. doi: 10.1037/amp0000825, 34410743

[68] Yeh CJ Inman AG Kim AB Okubo Y. Asian American families’ collectivistic coping strategies in response to 9/11. Cult Divers Ethn Minor Psychol. (2006) 12:134–48. doi: 10.1037/1099-9809.12.1.134, 16594860

[69] Chen R. Weaving individualism into collectivism: Chinese adults’ evolving relationship and family values. J Comp Fam Stud. (2015) 46:167–79. doi: 10.3138/jcfs.46.2.167

[70] Lu L Kao S-F. Traditional and modern characteristics across the generations: similarities and discrepancies. J Soc Psychol. (2002) 142:45–59. doi: 10.1080/00224540209603884 11913834

[71] Davidsen AS. Phenomenological approaches in psychology and health sciences. Qual Res Psychol. (2013) 10:318–39. doi: 10.1080/14780887.2011.608466, 23606810 PMC3627202

[72] Yang JP Do QA Nhan ER Chen JA. A mixed-methods study of race-based stress and trauma affecting Asian Americans during COVID. Clin Psychol Sci. (2024) 12:468–85. doi: 10.1177/21677026231180810, 37578208 PMC10345399

[73] Sabharwal M Becerra A Oh S. From the Chinese exclusion act to the COVID-19 pandemic: a historical analysis of “otherness” experienced by Asian Americans in the United States. Public Integr. (2022) 24:535–49. doi: 10.1080/10999922.2022.2120292

[74] Yan X Zhu Y Hussain SA Bresnahan M. Anti-Asian microaggressions in the time of COVID-19: impact on coping, stress, and well-being. Asian Am J Psychol. (2022) 13:248–58. doi: 10.1037/aap0000281

[75] Donnelly R Farina MP. How do state policies shape experiences of household income shocks and mental health during the COVID-19 pandemic? Soc Sci Med. (2021) 269:113557. doi: 10.1016/j.socscimed.2020.113557, 33308909 PMC7781085

[76] Donnelly R Frierson W Zhang Z Boen CE. State-level paid sick leave policies and population mental health during the COVID-19 pandemic in the United States. Soc Ment Health. (2024):21568693241300133. doi: 10.1177/21568693241300133 PMC12360710 40837275

[77] Azhar S Farina A Alvarez ARG Klumpner S. Asian in the time of COVID-19: creating a social work agenda for Asian American and Pacific islander communities. Soc Work. (2022) 67:58–68. doi: 10.1093/sw/swab044, 34747473

[78] Lu Y Wang C. Asian Americans’ racial discrimination experiences during COVID-19: Social support and locus of control as moderators. Asian American Journal of Psychology. (2022) 13:283.

